# A cross-sectional study examining convergent validity of a frailty index based on electronic medical records in a Canadian primary care program

**DOI:** 10.1186/s12877-019-1119-x

**Published:** 2019-04-16

**Authors:** Marjan Abbasi, Sheny Khera, Julia Dabravolskaj, Ben Vandermeer, Olga Theou, Darryl Rolfson, Andrew Clegg

**Affiliations:** 1 Department of Family Medicine, University of Alberta, Suite 205 College Plaza, 8215 - 112 St, Edmonton, AB T6G 2C8 Canada; 2 School of Public Health, University of Alberta, 8303 112 St NW, Edmonton, AB T6G 2T4 Canada; 3 Department of Pediatrics, University of Alberta, Edmonton Clinic Health Academy, 11405-87 Avenue, Edmonton, AB T6G 1C9 Canada; 4 Department of Medicine, Dalhousie University, 5955 Veterans’ Memorial Lane, Rm 1313, CHVMB, Halifax, B3H2E1 Nova Scotia Canada; 5 Department of Medicine, Division of Geriatric Medicine, University of Alberta, 1-198 Clinical Sciences Building, 11350-83 Avenue, Edmonton, AB T6G 2P4 Canada; 6 NIHR CLAHRC Older People’s Theme Academic Unit of Elderly Care and Rehabilitation, University of Leeds, Bradford Institute for Health Research, Temple Bank House, Bradford Royal Infirmary, Duckworth Lane, Bradford, BD9 6RJ UK

**Keywords:** Frailty case-finding, Electronic frailty index, Primary care

## Abstract

**Background:**

An electronic frailty index (eFI) has been developed and validated in the UK; it uses data from primary care electronic medical records (EMR) for effective frailty case-finding in primary care. This project examined the convergent validity of the eFI from Canadian primary care EMR data with a validated frailty index based on comprehensive geriatric assessment (FI-CGA), in order to understand its potential use in the Canadian context.

**Methods:**

A cross-sectional validation study, using data from an integrated primary care research program for seniors living with frailty in Edmonton, AB. Eighty-five patients 65 years of age and older from six primary care physicians’ practices were recruited. Patients were excluded if they were under 65 years of age, did not provide consent to participate in the program, or were living in a long term care facility at the time of enrolment. We used scatter plots to assess linearity and Pearson correlation coefficients to examine correlations.

**Results:**

Results indicate a strong statistically significant correlation between the eFI and FI-CGA (r = 0.72, 95% CI 0.60–0.81, *p* < 0.001). A simple linear regression showed good ability of the eFI scores to predict FI-CGA scores (F (1,83) = 89.06, *p* < .0001, R2 = 0.51). Both indices were also correlated with age, number of chronic conditions and number of medications.

**Conclusions:**

The study findings support the convergent validity of the eFI, which further justifies implementation of a case-finding tool that uses routinely collected primary care data in the Canadian context.

## Background

Approximately 1 in 4 older adults presenting to primary care are living with frailty and face the threat of declining health, poor quality of life, loss of independence, and greater reliance on higher levels of care [1]. Frailty is a state of increased vulnerability to stressors involving loss of reserves in interrelated biological, psychological and social domains [2–4]. Due to the detrimental impact of frailty and the potential to mitigate its adverse health outcomes with targeted interventions [5], international consensus guidelines recommend case-finding of frailty in primary care as part of routine clinical practice [6–8]. However, a key difficulty in widespread implementation of this recommendation has been the reliance on opportunistic case-finding in clinical practice using bedside instruments and questionnaires. Many of these tools require additional time, training, use of specialized equipment, or clinical resources, thus hindering efficiency and consistency in a busy primary care setting.

A recent breakthrough in the United Kingdom (UK) has been the development, validation and national implementation of an electronic frailty index (eFI) for frailty case-finding in primary care using data from electronic medical records [9]. The eFI is based on the deficit accumulation approach to frailty. This approach, developed first by Rockwood, Mitnitski and colleagues [10], identifies frailty based on a range of variables (e.g., signs, symptoms, diseases, disabilities, impairments, abnormal test values) collectively referred to as health deficits [11]. According to this model, frailty can be measured by calculating a frailty index (FI) that can be generated from any appropriately populated healthcare database [12–14] provided that there are a sufficient number of health deficits that satisfy certain criteria [13, 15]. Primary care electronic medical records (EMRs) contain rich data on a patient’s health and psychosocial context that make it a promising dataset to generate a FI score. The eFI in UK has been developed and validated using routinely available primary care EMR data from around 900,000 patients [9]. A pilot study in primary care in England demonstrated that the eFI was simple and quick to use (i.e. it is fully automated and scores are available at point of care), acceptable to practice staff, and was able to discriminate older patients referred for comprehensive geriatric assessment (CGA) from the total practice population [16].

While there is a number of frailty indices developed so far, the eFI represents an interesting approach of using routinely available data in the primary care EMR for frailty identification. However, there is limited knowledge of the applicability and validity of such FI in the Canadian context. In this project, the convergent validity—a core component of construct validity of a test [17]—of the eFI (calculated manually) from Canadian primary care EMR data with a validated frailty index tool, a frailty index based on a comprehensive geriatric assessment (FI-CGA) was examined.

## Methods

### Study design, setting and subjects

This cross-sectional study included 85 patients from the Seniors’ Community Hub (SCH), located in Edmonton, Alberta, Canada. The SCH is a team-based, integrated primary care research program guided by a geriatric specialist that aims to improve care for seniors living with frailty in the community. Patients 65 years of age and older from six primary care physicians’ practices were assessed in the SCH. Patients were excluded if they were under 65 years of age, did not provide consent to participate in the SCH, or were living in a long term care facility at the time of enrollment. These 85 patients received a CGA by a geriatric assessment nurse who was trained by the geriatric specialist. Twelve participants were seen by the geriatric specialist but did not consent to participation in the study and hence no data on them were collected.

### Frailty measurements

#### eFI

The eFI includes 36 health deficits derived from the EMR (comorbidities, physical impairments, clinical signs, symptoms, abnormal test values, and social circumstances; see Appendix 1) [9] EMRs in Canada were developed for transactional patient management rather than reporting and much of the data is in narrative/open text form. Multiple studies have shown that the combination of structured and unstructured data (International Classification of Diseases 9th revision codes, information about medications, laboratory values, visit notes) in EMR results in the best performance for disease identification [18, 19]. Therefore, a trained research assistant *manually* calculated the eFI scores from patient EMR using all available data sources (e.g. billing and diagnostic codes, problem list, medication list, and free text (visit notes)). As such, this process differs from the eFI validated in the UK where the 36 deficits are linked to over 2000 Read codes and retrieved automatically if present within the primary care EMR system. The research assistant coded deficits as 1 if present in the EMR data for that patient; and 0 if absent, whether the patient does not have that deficit or this information is missing in the EMR. All structured and unstructured data available starting 2012 (i.e. when the EMR was implemented in the participating clinic) was used for eFI calculation. For example, if chronic kidney disease was mentioned in 2013, the corresponding eFI deficit was checked off. However, for temporary conditions (e.g. anemia) the “look-back” period was one year. The eFI score was calculated by dividing the total number of deficits present by 36.

#### Fi-CGA FI-CGA

CGA is the current criterion standard for frailty identification and management [20, 21]. CGA is defined here as a thorough interdisciplinary and multidimensional assessment process used to determine the medical, functional, social, and psychological aspects of an older adult living with frailty and guide individualized care and support planning [22]. As with a primary care derived FI, a frailty index can also be derived ‘a posteriori’ from the content of the CGA (FI-CGA).

FI-CGA has been previously developed and validated based on its ability to predict individuals at higher risk of adverse health outcomes [23]. We constructed an FI-CGA that included 41 variables (see Appendix 2) following a standard protocol [13] from the CGA completed as part of the SCH process of care. The health deficits included in the FI-CGA comprised ordinal and nominal variables: all continuous variables were transformed into categorical variables. Missing data could occur with a variable like gait speed if the patient was unable to walk. Any patient who was missing 20% or more of the variables were excluded from the study. A family physician (member of the research team who is well versed in the CGA) independently calculated FI-CGA scores for all patients based on the content of the completed CGAs. FI-CGA scores were calculated by taking the sum of the deficits present and dividing it by 41.

### Statistical analysis

Descriptive statistics were used for the sample population. We used scatter plots to assess linearity and Pearson correlation coefficients to examine convergent validity between eFI and FI-CGA, as well as to examine correlation between both indices and age, number of chronic conditions, and number of medications. We used the following interpretation of correlation coefficients: 0 to 0.19 = very weak; 0.20 to 0.39 = weak; 0.40 to 0.59 = moderate; 0.60 to 0.79 = strong; 0.80 to 1.0 = very strong [24]. The correlation analysis was followed by regression analysis to construct a best fit model to predict FI-CGA scores based on eFI scores. We used independent-samples t-test to assess differences in eFI and FI-CGA scores between different groups of participants (with grouping factors being sex, living status, number of chronic conditions, etc.). The level of statistical significance was set at an alpha level of 0.05. All statistical analysis was conducted using SAS 9.4 (SAS institute Inc., Cary NC, USA).

#### Ethics

The Health Research Ethics Board, University of Alberta approved the study, and each participant signed informed consent forms. Funding was received from the Covenant Health Network of Excellence in Seniors’ Health and Wellness, as specified in the Acknowledgment. The funding agency did not have any role in formulating the research question and objectives, conducting analysis or preparing the manuscript.

## Results

### Description of the sample

The sample (*n* = 85) consisted of 51 (60%) females and the mean age was 81.1 (Me = 82, SD = 7.6), with 60% being 80 years and older. Table 1 shows the main characteristics of the sample. None of the patients had more than 20% missing values. There were only two variables with missing values: body mass index (BMI) and 4 m walk test were missing in 3 (3.5%) and 8 (9.4%) patients, respectively. Although the frailty index is meant to be used as a continuous score [25], to describe different frailty levels as defined by the FI-CGA and eFI, we used proposed cut-off scores identified using stratum specific likelihood ratios by Hoover et al. [26] that had been validated in a sample of community dwelling seniors in Canada: non-frail (0 to ≤0.1), vulnerable (> 0.1 to ≤0.21), frail (> 0.21 to < 0.45), and most frail (> 0.45) [26]. However, due to low frequency of scores of 0.1 and less (only one person), we merged non-frail and vulnerable categories as following: non-frail (0 to ≤0.21), frail (> 0.21 to < 0.45), and most frail (> 0.45 ≥0.45). According to the eFI and FI-CGA scores, 12 (14.1%) and 15 (17.6%) were considered non-frail, 66 (77.6%) and 51 (60%) – frail, and 7 (8.2%) and 19 (22.4%) – most frail, respectively.

**Table 1:** Sample characteristics (*N* = 85)

Age, mean, M (Me, SD)	81.1 (82; 7.6)
Female, N (%)	51 (60)
Marital status, N (%):	
- Married/common-law partner	45 (52.9)
- Divorced/separated	5 (5.9)
- Single	8 (9.4)
- Widowed	27 (31.8)
Education, N (%):	
-no formal education	1 (1.2)
-primary school	16 (18.8)
-secondary school	38 (44.7)
-post-secondary school	30 (35.3)
Lives alone, N (%)	29 (34.1)
Use of formal home support, N (%)	23 (27.1)
Taking 5 and more medications (prescription and over the counter), N (%)	71 (83.5)
Having 3 and more chronic conditions, N (%)	76 (89.4)
Reason for assessment in SCH	
Cognitive impairment/dementia	27 (31.8)
Caregiver burden	10 (11.8)
Chronic pain	16 (18.8)
Depression	15 (17.6)
Failure to thrive	2 (2.4)
Falls and decreased mobility	26 (30.6)
Home support	1 (1.2)
Medication review or polypharmacy	10 (11.8)
Medically complex	9 (10.6)
Other (e.g. maintaining health, general fatigue, interest in the program)	25 (29.4)

#### Distribution of eFI and FI-CGA

**Fig. 1:**
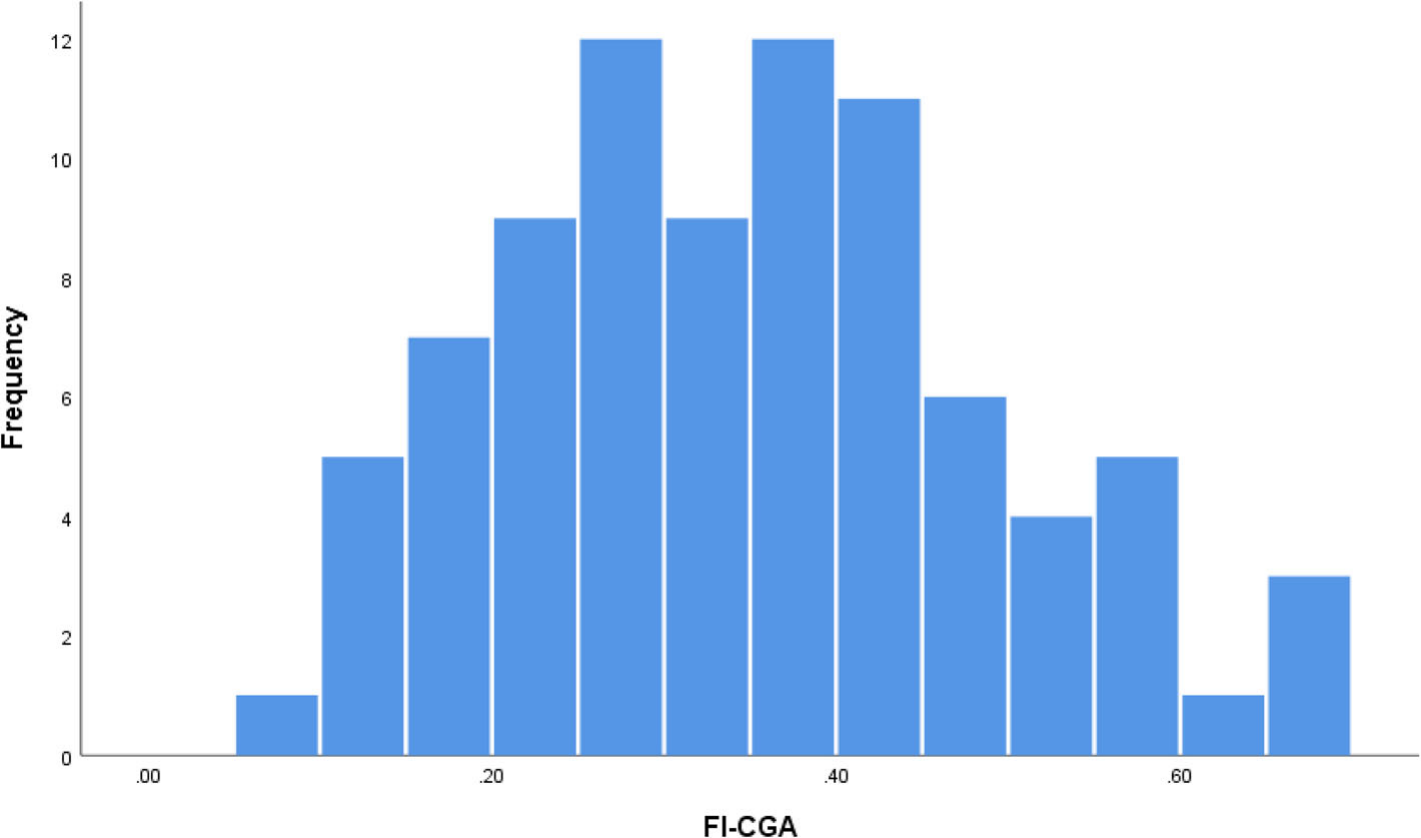
Distribution of FI-CGA scores

**Fig. 2:**
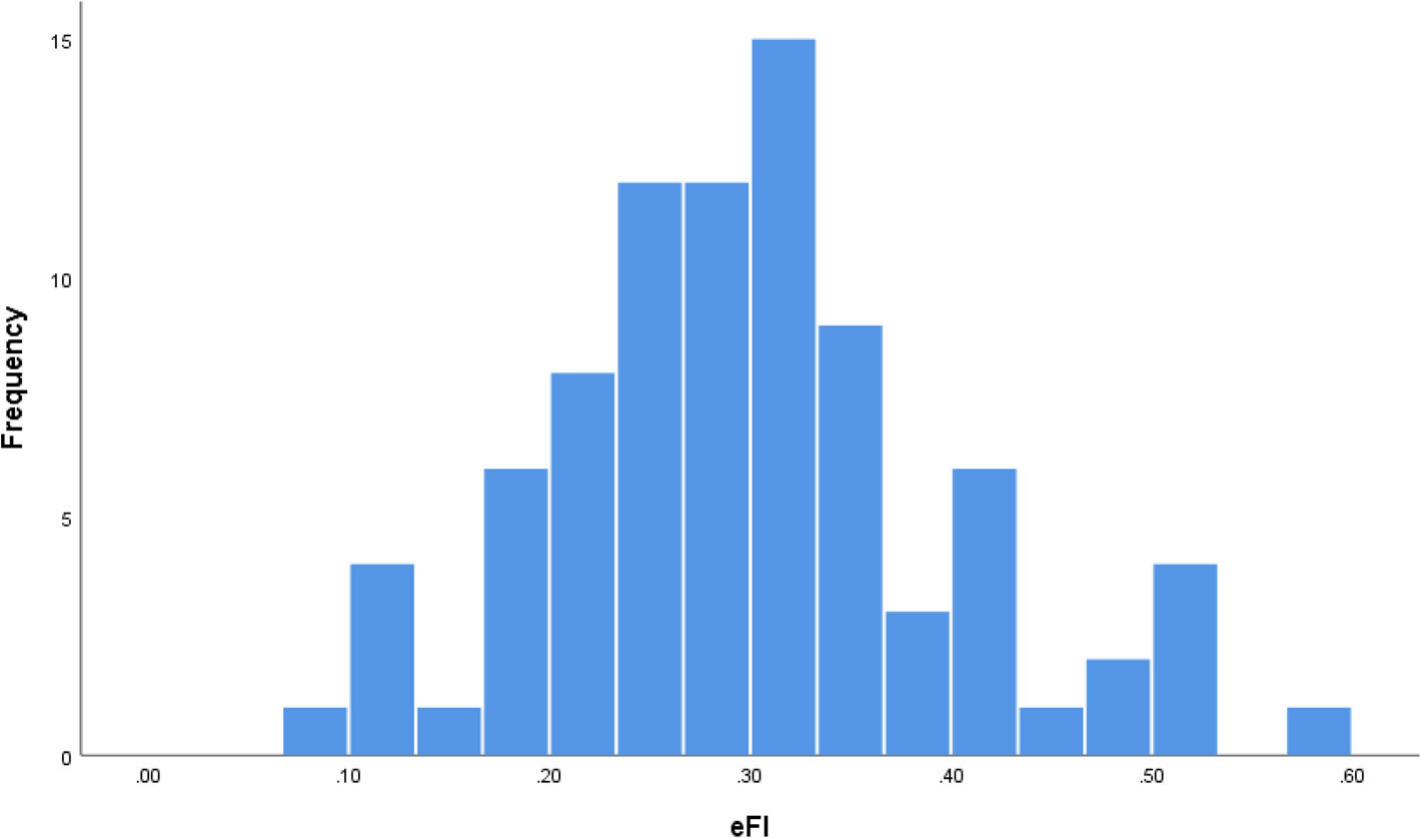
Distribution of eFI scores

Mean scores (and SD) for eFI and FI-CGA were 0.30 (0.10) and 0.35 (0.11), respectively. The difference in the mean scores was statistically significant (*p* < 0.001). The distribution of the FI-CGA and eFI scores approximates normal distribution (see Figs. 1 and 2, respectively). No ceiling or floor effects for FI-CGA (max = 0.69, min = 0.10) and eFI (max = 0.58, min = 0.08) were observed.

#### Correlation and regression analysis

**Fig. 3:**
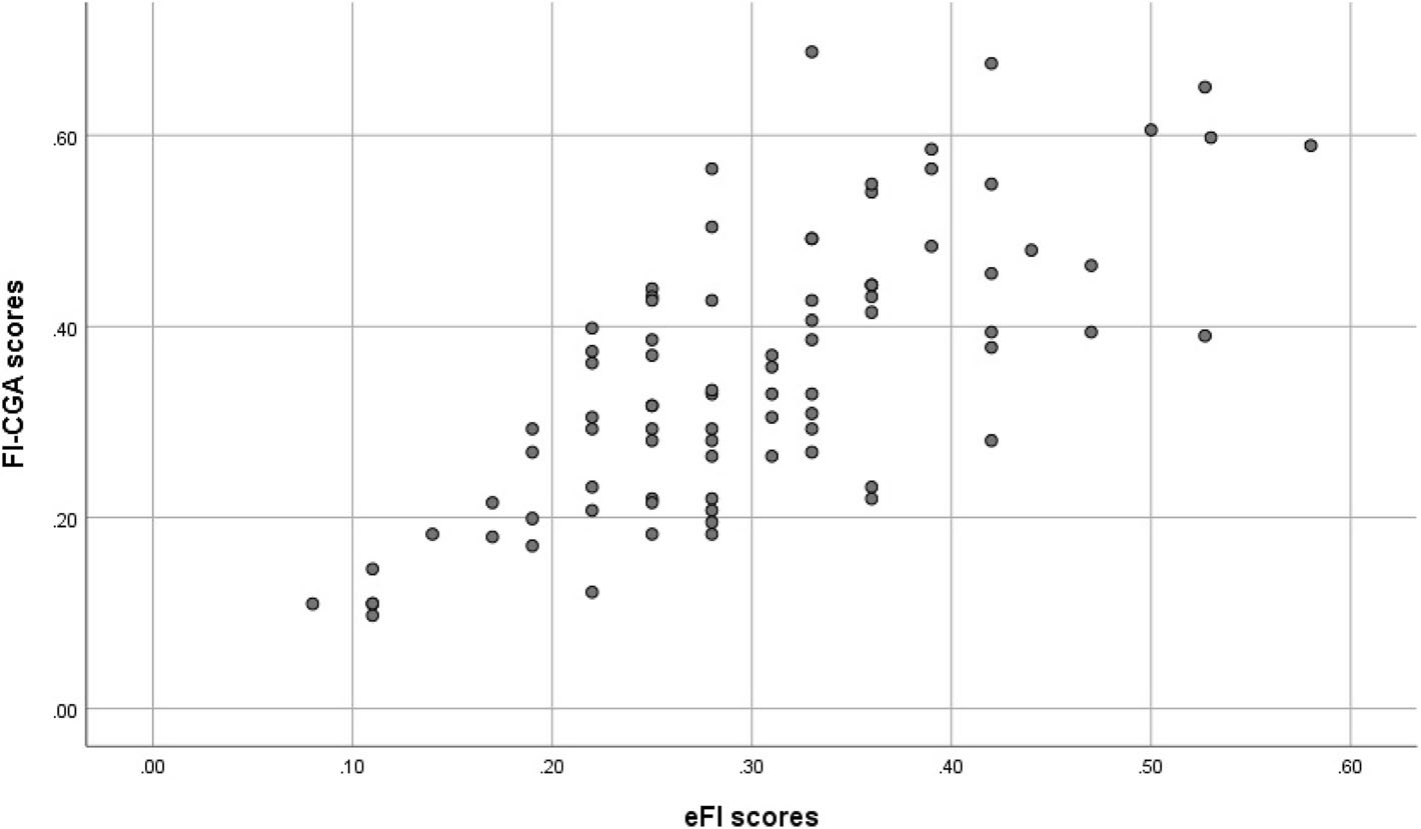
Scatterplot of the eFI and FI-CGA scores

The scatterplot depicting the relationship between the eFI and FI-CGA, which was best described using a linear model, is shown in Fig. 3. Results indicate strong statistically significant correlation between the eFI and FI-CGA (r = 0.72, 95% CI 0.60–0.81, p < 0.001).

A simple linear regression was calculated to predict FI-CGA scores based on eFI scores. A significant regression equation was found (F (1,83) = 89.06, *p* < .0001). Participants’ predicted FI-CGA score is equal to 0.994*eFI + 0.052. So, on average, eFI is FI-CGA shifted down by 0.052—of course there is variance from patient to patient, but the fit of the model was significant.

Both indices were also correlated with age, number of chronic conditions and number of medications (see Table 2). Overall, the degree of correlation between these indices and included factors was weak to moderate. However, the degree of correlation between eFI and aforementioned factors was higher compared to correlation between FI-CGA and these factors.

**Table 2:** Correlation of eFI and FI-CGA scores with age, number of chronic conditions, number of medications

	eFI, r (p)	FI-CGA, r (p)
Age	0.332 (0.002)**	0.226 (0.037)*
Number of chronic conditions	0.455 (< 0.001)**	0.331 (0.002)**
Number of medications	0.463 (< 0.001)**	0.418 (< 0.001)**

*p < 0.05; **p < 0.001

#### Description of eFI and FI-CGA scores in the examined sample

The difference in mean scores of eFI and FI-CGA in independent groups (depending on various grouping factors) is shown in Table 3. As measured by FI-CGA, women had higher levels of frailty than men (0.378 vs 0.315, respectively, *p* = 0.045); however, no significant difference was found in the levels of frailty as measured by eFI. Higher scores of both eFI and FI-CGA indices were observed in patients with 3 and more chronic conditions, polypharmacy, history of falls in the past 12 months and urinary incontinence. No significant difference in scores was found in those who live and do not live alone.

**Table 3:** eFI and FI-CGA scores as compared in different groups

	eFI scores, M (SD)	FI-CGA scores, M (SD)
Sex		
Males (*n* = 34)	0.30 (0.11)	0.32 (0.15)
Females (*n* = 51)	0.31 (0.10)	0.38 (0.14)
*p-value*	*0.627*	0.045*
Chronic conditions:		
Less than 3 (*n* = 9)	0.17 (0.06)	0.24 (0.12)
3 and more (*n* = 76)	0.32 (0.10)	0.37 (0.14)
*p-value*	< 0.001**	0.008*
Polypharmacy:		
Yes (*n* = 71)	0.32 (0.10)	0.38 (0.14)
No (*n* = 14)	0.23 (0.08)	0.24 (0.11)
*p-value*	0.002*	0.001*
Living alone:		
Yes (*n* = 29)	0.30 (0.09)	0.34 (0.11)
No (*n* = 56)	0.30 (0.11)	0.36 (0.16)
*p-value*	*0.995*	*0.541*
Falls in the past 12 months:		
Yes (*n* = 42)	0.35 (0.09)	0.42 (0.13)
No (*n* = 43)	0.26 (0.10)	0.29 (0.13)
*p-value*	< 0.001**	< 0.001**
Urinary incontinence:		
Yes (*n* = 28)	0.35 (0.09)	0.45 (0.11)
No (*n* = 57)	0.28 (0.10)	0.31 (0.14)
*p-value*	0.003*	< 0.001**

*p < 0.05; **p < 0.001

## Discussion

This is one of the first studies to investigate the relationship between the eFI and FI-CGA as different instruments designed to assess the construct of frailty as a state, and both applied to the Canadian primary care setting. The analysis demonstrated a linear relationship and strong correlation between the eFI and FI-CGA scores, with both lower and upper limits of the 95% confidence interval supporting this strong correlation. Thus, the study findings support the convergent validity of the eFI in relation to the FI-CGA, a core component of its construct validity.

The distribution of both scores approximated a normal distribution, which is expected in a population of oldest old (> 80 years old) who warranted CGA in a primary care setting. The distribution of the FI becomes less skewed as the mean age of the sample increases, and its relative heterogeneity diminishes [27]. In our study both indices were free of ceiling and floor effects, which is consistent with the results reported in other studies [28, 29] and might indicate that both indices were constructed with consideration of the selection criteria for health deficits outlined by Searle et al. [13] Proper deficit selection is crucial in establishing the consistent ability of the FI to determine frailty levels [28].

While strong correlation between two indices can be explained by the fact that they are based on the same theoretical framework of the cumulative deficit model of frailty, the results of the two indices were not in complete agreement. For example, the mean score for the eFI was significantly lower than that of the FI-CGA. This could be due to low prevalence or reporting of deficits derived from routine primary care data as reported elsewhere [30]. Reasons for this may include suboptimal data entry, tendency of patients not to discuss all of their health and social concerns during a clinic visit, and the greater emphasis on comorbidities rather than function, mobility and health attitudes in this dataset. As such, if frailty-related data is systematically missing from the record, it may be assumed incorrectly to be absent [30]. In our SCH cohort, the deficits were clearly defined and recorded as present if charted in the patient EMR, while eFI deficits not found in EMR were treated as absent. In contrast, the FI derived from the CGA intentionally explores and records challenging cognitive, psychosocial, and functional aspects of frailty and geriatric syndromes thus enriching the FI-CGA.

An important limitation was that the study sample was small; nevertheless, the 95% confidence interval was narrow, which indicates a statistically significant correlation even in such a small sample size. In addition, the sample consisted of community-dwelling older adults, not living in long-term care facilities, that were identified by family physicians as having ongoing concerns (in many cases, multiple concerns), and thus received an assessment by the SCH. This may limit the generalizability of the study findings to the very fit/robust or more functionally dependent or severely frail older adult populations. Other limitation is that the cross sectional design does not allow to compare the predictive performance of the eFI and FI-CGA. Future research may consider the predictive ability of the eFI generated from Canadian Primary Care EMR before being widely implemented. Another important limitation of this study was that the eFI calculation relied on labour-intensive review of medical records, which defeats the fundamental purpose of the eFI for rapid frailty case-finding in primary care. Since much of the data is in narrative/open text form in Canadian Primary Care primary care EMRs, innovative technologies in computer science such as Natural Language Processing and Machine Learning could facilitate the future automation of the eFI in primary care.

Results indicated that measured by FI-CGA, women had higher levels of frailty than men. This finding is not new and confirmed by numerous studies [1, 26, 31, 32]. Moreover, Herr et al. [33], in their study of life expectancy in the state of frailty, also point to the fact that women live longer despite bearing a larger burden of health deficits than men. The authors explain this sex difference as an interplay of various social, behavioral, and biological factors. There was no difference in the eFI scores based on sex, which could be explained by the deficits included in the eFI; the comorbidities are more prevalent than functional measures.

Regardless of these differences, both indices correlated with age, number of chronic conditions and number of medications. In both the eFI and the FI-CGA, the strength of association was weak to moderate. However, the strength of these correlations was higher in the eFI, which may reflect its greater dependence on these deficits compared to the FI-CGA which includes additional assessment information, reflecting the complex and rich nature of frailty. Similar to other studies based on the FI [30, 34], higher scores of both eFI and FI-CGA indices were observed in patients with 3 and more chronic conditions, polypharmacy, history of falls in the past 12 months and urinary incontinence. No significant difference in scores was found in those living alone. However, this finding should be interpreted with caution, as living alone does not mean having no social support, nor does it exclude the possibility of ‘assets’ that make such an individual more resilient.

## Conclusion

This study supports the convergent validity of the eFI with the FI-CGA. Health deficits that comprise the eFI in the UK were captured in the Canadian primary care EMR. The findings demonstrated a strong, linear association with the validated frailty index, FI-CGA. Therefore, the eFI has the potential to be implemented in Canada as a case-finding instrument. More research is needed to understand its performance in Canadian EMR data and in a more representative community sample.

## Appendix 1

**Table 4:** 36 health deficits included in the eFI

*Diseases*	*Functional abilities*
Arthritis Atrial fibrillation Cerebrovascular disease Chronic kidney disease COPD Diabetes Foot problems Fragility fracture Heart failure Heart valve disease Hypertension Hypotension/syncope Ischemic heart disease Osteoporosis Parkinsonism and tremor Peptic ulcer Peripheral vascular disease Respiratory disease Skin ulcer Thyroid disease	Dizziness Dyspnea Falls Memory and cognitive problems Polypharmacy Sleep disturbance Urinary incontinence Weight loss and anorexia
	*Disabilities*
	Activity limitation Hearing impairment Housebound Mobility and transfer problems Requirement for care Social vulnerability Visual impairment
	*Labs*
	Anemia and hematinic deficiency

## Appendix 2

**Table 5:** Variables included in the FI-CGA

Variable	Coding
1. Body mass index (BMI)	Normal (BMI from 18.5 to 25) = 0; Abnormal (BMI less than 18.5 or more than 25) = 1
2. 4-m walk test	Less than 5 s = 0; 5 s and more = 1
3. Formal home support	No = 0; Yes = 1
4. Hypertension	No = 0; Yes = 1
5. CHF	No = 0; Yes = 1
6. CAD	No = 0; Yes = 1
7. Arrhythmias	No = 0; Yes = 1
8. Hyperlipidemia	No = 0; Yes = 1
9. Stroke or TIA	No = 0; Yes = 1
10. Arthritis	No = 0; Yes = 1
11. Asthma	No = 0; Yes = 1
12. Cancer	No = 0; Yes = 1
13. COPD	No = 0; Yes = 1
14. Diabetes	No = 0; Yes = 1
15. Number of medications (prescription and over-the-counter)	Less than 4 = 0; 4–7 = 0.5; More than 7 = 1
16. Bathing	Independent = 0; With assistance = 0.5; Dependent = 1
17. Toileting	Independent = 0; With assistance = 0.5; Dependent = 1
18. Dressing	Independent = 0; With assistance = 0.5; Dependent = 1
19. Feeding	Independent = 0; With assistance = 0.5; Dependent = 1
20. Cooking	Independent = 0; With assistance = 0.5; Dependent = 1
21. Shopping	Independent = 0; With assistance = 0.5; Dependent = 1
22. Cleaning	Independent = 0; With assistance = 0.5; Dependent = 1
23. Meds	Independent = 0; With assistance = 0.5; Dependent = 1
24. Driving	Independent = 0; With assistance = 0.5; Dependent = 1
25. Banking	Independent = 0; With assistance = 0.5; Dependent = 1
26. Walking	Independent = 0; With assistance = 0.5; Dependent = 1
27. Transfers	Independent = 0; With assistance = 0.5; Dependent = 1
28. Aids	None = 0; Cane = 0.33; Walker = 0.66; Wheelchair = 1
29. Falls in the past 12 months	No = 0; Yes = 1
30. Cognition	No = 0; Concerns from family or mild cognitive impairment = 0.5; Dementia diagnosis = 1
31. Mood	No = 0; Low mood but does not meet criteria for depression = 0.5, Depression = 1
32. Anxiety	No = 0; Yes = 1
33. Delusions/hallucinations	No = 0; Yes = 1
34. Vision impairment	No = 0; Functional with aid = 0.5; Yes = 1
35. Hearing impairment	No = 0; Functional with aid = 0.5; Yes = 1
36. Weight loss	No or intentional weight loss = 0; Yes = 1
37. Bladder incontinence	No = 0; Yes = 1
38. Bowel incontinence	No = 0; Yes = 1
39. Chronic pain	No = 0; Yes = 1
40. Physical activity	No = 1; Yes = 0
41. Bone health	No history of problems = 0; History of osteoporosis, fractures etc. = 1

## Abbreviations

BMI: Body mass index
CGA: Comprehensive geriatric assessment
CI: Confidence interval
eFI: Electronic frailty index
EMR: Electronic medical record
FI: Frailty index
FI-CGA: Frailty index based on a comprehensive geriatric assessment
M: Mean
Me: Median
SCH: Seniors’ Community Hub
SD: Standard deviation
UK: United Kingdom
