# Correction to: A cross-sectional study examining convergent validity of a frailty index based on electronic medical records in a Canadian primary care program

**DOI:** 10.1186/s12877-019-1144-9

**Published:** 2019-05-13

**Authors:** Marjan Abbasi, Sheny Khera, Julia Dabravolskaj, Ben Vandermeer, Olga Theou, Darryl Rolfson, Andrew Clegg

**Affiliations:** 1 Department of Family Medicine, University of Alberta, Suite 205 College Plaza, 8215 - 112 St, Edmonton, AB T6G 2C8 Canada; 2 School of Public Health, University of Alberta, 8303 112 St NW, Edmonton, AB T6G 2T4 Canada; 3 Department of Pediatrics, Edmonton Clinic Health Academy, University of Alberta, 11405-87 Avenue, Edmonton, AB T6G 1C9 Canada; 4 Department of Medicine, Dalhousie University, 5955 Veterans’ Memorial Lane, Rm 1313, CHVMB, Halifax, Nova Scotia B3H2E1 Canada; 5 Department of Medicine, Division of Geriatric Medicine, University of Alberta, 1-198 Clinical Sciences Building, 11350-83 Avenue, Edmonton, AB T6G 2P4 Canada; 6 NIHR CLAHRC Older People’s Theme Academic Unit of Elderly Care and Rehabilitation, Bradford Institute for Health Research, Temple Bank House, Bradford Royal Infirmary, University of Leeds, Duckworth Lane, Bradford, BD9 6RJ UK

**Correction to: BMC Geriatr**

**https://doi.org/10.1186/s12877-019-1119-x**

Following the publication of this article [1], the authors reported an error in the “Results” section. The error occurred in the sentences:

Although the frailty index is meant to be used as a continuous score [25], to describe different frailty levels as defined by the FI-CGA and eFI, we used proposed cut-off scores identified using stratum specific likelihood ratios by Hoover et al. [26] that had been validated in a sample of community dwelling seniors in Canada: non-frail (0 to ≤0.1), vulnerable (> 0.1 to ≤0.21), frail (> 0.21 to < 0.45), and most frail (> 0.45) [26]. However, due to low frequency of scores of 0.1 and less (only one person), we merged non-frail and vulnerable categories as following: non-frail (0 to ≤0.21), frail (> 0.21to < 0.45), and most frail (> 0.45 ≥0.45).

The final value for both sentences should in fact read: “most frail (≥0.45).”

The original article has been corrected, and the publisher apologizes to the authors and readers for any inconvenience.
